# Using internet enabled mobile devices and social networking technologies to promote exercise as an intervention for young first episode psychosis patients

**DOI:** 10.1186/1471-244X-11-80

**Published:** 2011-05-12

**Authors:** Eoin Killackey, Anna Lee Anda, Martin Gibbs, Mario Alvarez-Jimenez, Andrew Thompson, Pamela Sun, Gennady N Baksheev

**Affiliations:** 1 Orygen Youth Health Research Centre, Centre for Youth Mental Health, The University of Melbourne, Locked Bag 10, 35 Poplar Rd, Parkville, Victoria 3052, Australia; 2 Deparment of Information Systems, The University of Melbourne, 111 Barry St, Carlton, Victoria 3010, Australia

## Abstract

**Background:**

Young people with first episode psychosis are at an increased risk for a range of poor health outcomes. In contrast to the growing body of evidence that suggests that exercise therapy may benefit the physical and mental health of people diagnosed with schizophrenia, there are no studies to date that have sought to extend the use of exercise therapy among patients with first episode psychosis. The aim of the study is to test the feasibility and acceptability of an exercise program that will be delivered via internet enabled mobile devices and social networking technologies among young people with first episode psychosis.

**Methods/Design:**

This study is a qualitative pilot study being conducted at Orygen Youth Health Research Centre in Melbourne, Australia. Participants are young people aged 15-24 who are receiving clinical care at a specialist first episode psychosis treatment centre. Participants will also comprise young people from the general population. The exercise intervention is a 9-week running program, designed to gradually build a person's level of fitness to be able to run 5 kilometres (3 miles) towards the end of the program. The program will be delivered via an internet enabled mobile device. Participants will be asked to post messages about their running experiences on the social networking website, and will also be asked to attend three face-to-face interviews.

**Discussion:**

This paper describes the development of a qualitative study to pilot a running program coupled with the use of internet enabled mobile devices among young people with first episode psychosis. If the program is found to be feasible and acceptable to patients, it is hoped that further rigorous evaluations will ultimately lead to the introduction of exercise therapy as part of an evidence-based, multidisciplinary approach in routine clinical care.

## Background

Young people experiencing their first episode psychosis are at an increased risk of poor health outcomes, such as weight gain [1], cardiovascular disease and diabetes [2,3], and often present with significant medical co-morbidities [4]. Poor dietary choices, infrequent physical activity, and side effects from antipsychotic medications are some of the factors that are thought to account for the elevated levels of physical morbidity among this population [5,6]. Others have gone further to suggest that it is the general health and mental health systems that have shown a lack of concern about the physical health of people with mental illness [7]. While physical exercise has surprisingly been a neglected intervention in mental health treatment packages [8], there has been a growing recognition that physical activity can aid in the process of psychiatric rehabilitation [9], with potential benefits for both physical and mental health [10].

The effectiveness of exercise as an adjunct therapy for established psychotic disorders was the subject of a recent critical review that showed good support for the improved mental health among participants following an exercise intervention [11]. While the quantitative findings were relatively weak, partly due to the small numbers of participants, findings from the qualitative interviews were more positive. Participants reported more positive psychological well-being and social interaction with family and friends following the intervention [12,13]. These findings are bolstered by randomised controlled trials that found significantly greater reductions in body fat [14] and significant improvements in depression, anxiety [10,15] and negative symptom scores [10,14] among experimental participants compared to controls. One trial compared yoga therapy with exercise therapy and found that while yoga therapy participants showed significantly reduced levels of psychopathology and greater quality of life when compared to physical exercise participants, both groups demonstrated an increase in social and occupational functioning [10,16].

No studies to date however, to the knowledge of the authors, have specifically investigated the physical and mental health benefits of exercise therapy among young people with first episode psychosis. Exercise Interventions in the early course of psychosis may lead to marked gains in health benefits for these young people [17]. Given the popularity of the internet among young people, the current study will pilot the use of internet enabled mobile devices to assist young people with mental illness to engage in a publicly available exercise program called *Couch to 5K *(C25K), which may assist in overcoming geographical barriers in attending regular onsite exercise classes [15]. The study will also utilise social network technologies to overcome motivational barriers in increasing and maintaining physical activity in everyday life [18]. Social interaction is important for an individual's wellbeing, and social networks provide the means to link individuals together to create a group dynamic and facilitate social interaction [19]. These social interactions between individuals and their influences on each other on the social network website may also facilitate behaviour change in relation to jogging, as the technology will allow other individuals to see when others are jogging, for how long and how far, encouraging others to exercise and be active also. This field of persuasive techniques is known as persuasive technology, which has been defined as technology that is intentionally designed to change a person's attitude or behaviour [20,21]. Persuasive technology can have a positive impact on the fitness of young people [22], and may assist in circumventing the poor health outcomes among patients with first episode psychosis.

The overall aim of this study is to develop a prototype and test services delivered through internet enabled mobile devices and social network technologies that are designed to encourage young people to engage in physical exercise as well as act as an intervention for weight gain. The study will also seek to investigate if the delivery of an exercise program via this medium would be acceptable to young people.

## Methods

### Study design

The study is a qualitative pilot study that is designed to explore the feasibility and acceptability of using internet enabled mobile devices and social network technologies in promoting exercise among young people. The study has been approved by Melbourne Health Human Research Ethics Committee.

### Setting

Orygen Youth Health (OYH) is a specialist public mental health service for people aged 15-24 years living in the Western and Northern regions of Melbourne. The service treats young people presenting with psychotic and non-psychotic disorders. The catchment area covers a population of approximately one million people. The study will take place in the Early Psychosis Prevention and Intervention Centre (EPPIC), a clinical program at Orygen Youth Health. EPPIC was established in 1992 with the aim of reducing the delay in detecting and treating psychosis and advocates for timely and comprehensive treatment during the early years following illness onset [23]. All people aged between 15 and 24 who are living in the catchment area and experiencing a first episode of psychosis are referred to this service and may receive clinical care for a period of 18 months.

### Participants

Inclusion criteria for the study include: (1) being a current client at EPPIC; and, (2) express an interest in participating in a running program, to ensure that they will complete the program and are willing to volunteer their time. It is anticipated that 10 young people will be recruited to the study. Participants will be excluded if they: (1) are not fluent in English, as participants will be asked to communicate their experiences of the running program with other participants; (2) are pregnant women; (3) have significant medical conditions for which they receive regular medical care, as this program will require participants to exercise three times a week, mostly outdoors; (4) have not been medically cleared for participating in a running program by a general practitioner (GP); and, (5) are in the acute phase of a psychotic illness.

Participants will also comprise 10 young people from the general population, to ascertain the feasibility of the running program with the use of internet enabled mobile devices among a selected sample of healthy controls. Inclusion criteria will include expressing an interest in participating in the running program. Exclusion criteria included: (1) not being fluent in English; (2) pregnant women; and, (3) having significant medical conditions. Participants will be recruited through friends and acquaintances of the research team.

### Materials

The running program will be delivered via an application implemented on an internet enabled mobile device, an iPod touch. The iPod touch is a product of Apple Inc, which incorporates a portable media player, a mobile Wi-Fi platform and a multi-touch graphical user interface. Two freely available applications will be downloaded onto the iPod touch for participants, the Couch to 5 k (C25K) application and the Nike+ iPod application. The C25K application will be utilised for guiding participants through the running program via audio cues, and will allow participants to listen to a playlist of songs of their choosing while completing their exercise. The Nike+ iPod application will be utilised to measure running activities from the Nike+ running sensor that will be attached to the participant's running shoe. The Nike+ sensor is similar to a pedometer that counts each step a person takes and calculates the distance travelled, duration of each run (time) and pace. Thus, the sensor can also act as a measure of program adherence by noting whether participants complete the scheduled exercises. Participants will also be provided with a sports armband to secure the iPod touch to their arm during the exercise, computer cables and earphones.

Participants will also be provided with a number of de-identified usernames and passwords with which they will be able to access the social networking website (i.e., Twitter), their email account, and Nike+ account. De-identified names will be used to ensure participants feel comfortable when writing about their running experiences and to protect their privacy. These will be set up specifically for the purpose of this research.

### Procedure

Members of the research team will raise awareness of the running program among EPPIC case managers by attending clinical meetings and distributing study brochures. Case managers will then be invited to refer suitable clients who might be interested in participating in the running program to the research team. Potential participants will meet with a Research Assistant (RA) who will explain all aspects of the study protocol except for one: that there is funding available to those who do not own a suitable pair of running shoes. It was decided not to inform all participants about this at the outset to avoid enticing people into participating for the wrong reasons. However, if a participant indicates that they would like to take part but are unable to because they do not have suitable running shoes, the RA would offer the participant support to purchase a pair of running shoes. Providing a good pair of running shoes to participants was considered important as this would play a significant role in minimising injuries from running and removing barriers to participation.

If the young person expresses an interest in taking part, the RA will obtain written consent from participants and their parent/guardian, where appropriate. Participants will also be provided with written information that explains the study details and the contact details of the research team. Mental state and capacity to provide consent will be determined both by the participant's case manager/doctor who are independent of the study and also by clinically experienced members of the research team.

Participants will be recruited at approximately the same time to form a virtual, co-located cohort of participants. Having provided their consent, participants will be shown how to use the technological components of the study, will be shown how to jog correctly if this is needed, and how to complete stretches before and after exercise. Given that young people experiencing first episode psychosis often present with significant medical co-morbidities [4], they will be screened for physical health conditions prior to starting the program by a general practitioner (GP). This was conducted to ensure that the intervention does not pose an unnecessary risk to participants. The GP will examine their medical history, prescribed medications, and general health status, paying particular attention to respiratory problems, diabetes and cardiac problems.

Participants will then take part in the 9-week running program (see below). All of the three weekly running sessions will be conducted by participants at home. Participants will be asked to spend approximately 5 minutes after each running session posting a 'blog' (i.e., a commentary or message) about their running experience to the social networking website, highlighting their successes and struggles during the session. Participants will also be asked to upload their results from the Nike+ sensor to their Nike+ account. Participants will also be advised that they can further interact with others on the social networking website. The information posted on the websites will only be available to participants in the study and members of the research team.

Participants will be asked to attend three face-to-face interviews during the course of the study. The first interview will take place at the beginning of the program, where participants will be queried about their aims for participating in the program, their current fitness habits, barriers and motivators for exercising, and physical health status. Another interview will be conducted at week 4 of the running program, with questions about progress with the program to date and what they like/dislike about the program and the technological components. The third interview will be conducted on completion of the running program at week 9, with questions about whether their aims for participating in the program were met and if they had noticed changes in their health. Interviews will be conducted individually with a member of the research team at a place of convenience for the participant, such as their home or at Orygen Youth Health. Interviews will be approximately 30 minutes in duration, and will be recorded verbatim utilising the Audacity^® ^1.2.6 program for Mac. Consent will be sought from each participant to record the interview, and will be stored electronically using the assigned identification number for each participant.

A similar procedure will be applied to the general population participants, except that running shoes will not be offered and neither will they be required to attend an appointment with the GP.

### Intervention

The Couch to 5 km program has been designed to get a person of any fitness level from the couch to running 5 kilometres in nine weeks. The Couch to 5 k program is comprised of three sessions per week. Each running session in turn is comprised of 30 minutes of alternating distances of walking and running, starting off with small distances of walking and running, and slowly building up to either running a distance of 5 kilometres (three miles) or a duration of 30 minutes towards the end of the program (See additional file 1: Couch to 5 k running program).

The Couch to 5 k program is a gentle introduction to getting the body moving, and eases people into the running program. The idea of the program is not to rush into running long distances from the start. Rather, the guiding philosophy of the program is to transform a person from being a 'couch potato' to a runner by slowing developing a person's fitness and strength and allowing the body to adjust to running over a 9-week period. Thus, while it may be tempting for people to skip ahead in the program and try and do more if they can, it is important that people keep to the running schedule to gradually build their level of fitness. On the other hand, if people find that the program is too strenuous, weeks can be repeated when needed until the person is comfortable with progressing to the following week. It is also important that the running sessions are spaced out throughout the week to give the body a chance to rest and recover between sessions. Interested readers can visit the Couch to 5 k website [24].

### Outcomes and assessments

The main outcomes of interest are whether or not the running program is found to be feasible among patients with first episode psychosis, and whether or not the program is found to be acceptable. The feasibility of the running program will be determined by assessing adherence to the structure of the running program, the frequency with which participants make use of the social networking technology, whether or not participants spend some time after each run communicating their experiences with other participants via twitter, and whether or not participants complete the running program. The acceptability of the running program will be determined by assessing what participants like/dislike about the features of the running program and the technological components, and the motivating factors for continuing with the running program as reported by participants in their 'blog' posts on twitter. Secondary outcomes of interest are whether or not participation in the running program leads to higher levels of self-reported fitness, and whether or not there is a relationship between the use of the social networking website and the up-take of the exercise intervention.

These outcomes will be assessed via the completed interviews at week 4 and also at week 9. The interviews at both time-points will include a series of questions designed to elicit whether or not participants like or dislike the running program and the use of the social networking technologies, whether any members of the group influenced their participation in the program, participants' motivations and barriers to engaging in the program, and any changes in their perceived health and well-being. Additional questions at week 9 will include whether the goals for participating in the program were met, if there were any other features that participants would have liked to be included in the program, and their thoughts and priorities about engaging in physical activity at the completion of the program.

Outcomes will also be assessed via the 'blogs' made to the social network website after each running session, the interactions between participants on the social network website, and also via the uploaded results from the Nike+ sensor.

### Data analysis

A range of descriptive statistics will be used to characterise the sample in relation to key demographic variables, such as age, gender, employment or educational status, and country of birth. The transcripts from the interviews, the interactions from the social network website and the 'blog' posts on this website will be observed and analysed using grounded theory [25] to examine the above-mentioned outcomes, such as whether or not participants find the running program feasible and the motivating factors that engage young people in exercise. Grounded theory is a widely used approach in qualitative research [26]. This research method is comprised of developing an integrated set of concepts that provide a thorough theoretical explanation of the social phenomena that is being studied [25]. Critical to this process is that the theory is grounded in the data, and explains the phenomena of habitual participation in exercise in terms of the conditions that facilitate this, its expression in action, and the consequences of these actions. To this end, coding will be the analytic process that will be used by the research team to arrive at these concepts. Open coding will be used at the beginning of the program, which is an interpretive process of breaking the data down into conceptual labels. Concepts will be compared against each other for similarities and differences, with similar concepts grouped together to create more abstract categories. By week 4 - 5 of the running program, axial coding will be implemented, which is where categories are related to their subcategories (i.e., elucidating the variations in a category under certain conditions), testing these relationships against data, and further developing other categories. Selective coding will be used towards the end of the running program, which is where categories are unified around a core category that represents the central phenomenon or the main analytic idea of the study [25]. Furthermore, data from the Nike+ sensor will be used to gauge how well participants adhere to the structure of the running program.

## Discussion

This paper describes the development of a qualitative study to pilot a running program coupled with the use of an internet enabled mobile device among young people experiencing their first episode of psychosis. While there is a growing body of evidence supporting the use of exercise therapy for physical and mental health benefits among people diagnosed with schizophrenia [10], there is currently a dearth of studies that have sought to extend exercise therapy as an adjunct intervention to young people experiencing their first episode of psychosis. There is also little evidence regarding the feasibility of using internet enabled mobile devices to promote exercise among this population. In addition, it is also hoped that this study will further elucidate the motivating factors for regular participation in exercise among this population.

Typically, clients at EPPIC are prescribed medication as part of their mental health treatment plans. However, antipsychotic medications have been recognised for inducing weight gain [27] and increasing metabolic syndrome indicators [28]. While studies have focussed on mitigating the weight gain side effects of antipsychotic medications among first episode psychosis patients [29], there is a critical need for studies to investigate how to increase participation in exercise among this population. The use of exercise therapy as an intervention may assist in improving the physical health status of this population, lead to a better quality of life, and also lead to a greater engagement with the community at large as patients will be in a position to participate in community-based activities such as running groups or fun runs.

If the research program is found to be feasible and acceptable to patients with first episode psychosis, the research team will seek funding to investigate the health benefits of this running program in a larger sample. Given that young people are increasing their usage of internet enabled mobile devices, and that these devices present new opportunities for the delivery of health services, it is important to rigorously examine how mobile devices might be incorporated and correctly utilised as an adjunct form of mental health intervention. Changing health behaviour for the long term requires maintaining a patient's motivation and interest over a protracted period of time [30]. The effective use of social networking to support such a behaviour change represents a significant gap in the current state of the research literature, particularly in relation to changing the exercise habits of patients with first episode psychosis. There appears to be a specific need for novel psychosocial interventions in addition to the use of medications in this population [31]; this study provides an avenue of possible research that uses technological components to promote exercise among patients with first episode psychosis. Ultimately, it is envisaged that exercise therapy may become part of routine clinical care as part of an evidence-based best practice multidisciplinary approach to mental healthcare.

## Competing interests

The authors declare that they have no competing interests.

## Authors' contributions

All authors have been involved in conception of the study, made substantial contributions to the study protocol, and drafting or revising the manuscript. All authors have read and approved the final manuscript.

## Pre-publication history

The pre-publication history for this paper can be accessed here:

http://www.biomedcentral.com/1471-244X/11/80/prepub

## Supplementary Material

Additional file 1 **Killackey et al C25k study protocol paper**. Click here for file
