# Supplementary material for: Using internet enabled mobile devices and social networking technologies to promote exercise as an intervention for young first episode psychosis patients
Source: BMC Psychiatry. 2011 May 12;11:80. doi: 10.1186/1471-244X-11-80 (PMC3118174; doi:10.1186/1471-244X-11-80)
Supplement: Additional file 1 — Killackey et al C25k study protocol paper. [file 1471-244X-11-80-S1.PDF]

Additional file 1: Couch to 5k running program

Week 1

Workout 1: Brisk five minute warm-up walk. Then alternate 60 seconds of jogging and 90 seconds of walking for a total of 20 minutes.
Workout 2: Brisk five minute warm-up walk. Then alternate 60 seconds of jogging and 90 seconds of walking for a total of 20 minutes.
Workout 3: Brisk five minute warm-up walk. Then alternate 60 seconds of jogging and 90 seconds of walking for a total of 20 minutes.

Week 2

Workout 1: Brisk five minute warm-up walk. Then alternate 90 seconds of jogging and two minutes of walking for a total of 20 minutes.
Workout 2: Brisk five minute warm-up walk. Then alternate 90 seconds of jogging and two minutes of walking for a total of 20 minutes.
Workout 3: Brisk five minute warm-up walk. Then alternate 90 seconds of jogging and two minutes of walking for a total of 20 minutes.

Week 3

Workout 1: Brisk five minute warm-up walk, then two repetitions of the following: Jog for 90 seconds; Walk for 90 seconds; Jog for 3 minutes; Walk for 3 minutes.
Workout 2: Brisk five minute warm-up walk, then two repetitions of the following: Jog for 90 seconds; Walk for 90 seconds; Jog for 3 minutes; Walk for 3 minutes.
Workout 3: Brisk five minute warm-up walk, then two repetitions of the following: Jog for 90 seconds, Walk for 90 seconds, Jog for 3 minutes; Walk for 3 minutes.

Week 4

Workout 1: Brisk five minute warm-up walk, then: Jog for 3 minutes, Walk for 90 seconds, Jog for 5 minutes, Walk for 2.5 minutes, Jog for 3 minutes, Walk for 90 seconds, Jog for 5 minutes.

Workout 2: Brisk five minute warm-up walk, then: Jog for 3 minutes, Walk for 90 seconds, Jog for 5 minutes, Walk for 2.5 minutes, Jog for 3 minutes, Walk for 90 seconds, Jog for 5 minutes.

Workout 3: Brisk five minute warm-up walk, then: Jog for 3 minutes, Walk for 90 seconds, Jog for 5 minutes, Walk for 2.5 minutes, Jog for 3 minutes, Walk for 90 seconds, Jog for 5 minutes.

Week 5

Workout 1: Brisk five minute warm-up walk, then: Jog for 5 minutes, Walk for 3 minutes, Jog for 5 minutes, Walk for 3 minutes, Jog for 5 minutes.

Workout 2: Brisk five minute warm-up walk, then: Jog for 8 minutes, Walk for 5 minutes, Jog for 8 minutes.

Workout 3: Brisk five minute warm-up walk, then jog for 20 minutes with no walking.

Week 6

Workout 1: Brisk five minute warm-up walk, then: Jog for 5 minutes, Walk for 3 minutes, Jog for 8 minutes, Walk for 3 minutes, Jog for 5 minutes.

Workout 2: Brisk five minute warm-up walk, then: Jog for 10 minutes, Walk for 3 minutes, Jog for 10 minutes.

Workout 3: Brisk five minute warm-up walk, then jog for 25 minutes with no walking.

Week 7

Workout 1: Brisk five minute warm-up walk, then jog for 25 minutes.

Workout 2: Brisk five minute warm-up walk, then jog for 25 minutes.

Workout 3: Brisk five minute warm-up walk, then jog for 25 minutes.

Week 8

Workout 1: Brisk five minute warm-up walk, then jog for 28 minutes.

Workout 2: Brisk five minute warm-up walk, then jog for 28 minutes.

Workout 3: Brisk five minute warm-up walk, then jog for 28 minutes.

Week 9

Workout 1: Brisk five minute warm-up walk, then jog for 30 minutes.

Workout 2: Brisk five minute warm-up walk, then jog for 30 minutes.

Workout 3: Brisk five minute warm-up walk, then jog for 30 minutes
